# Gender differences and cooperation in medical authorships - an analysis of the recent ten years in five key medical disciplines

**DOI:** 10.1186/s12909-023-04041-6

**Published:** 2023-01-27

**Authors:** Jin Yamamura, Isabel Molwitz, Ann-Kathrin Ozga, Thai-An Nguyen, Ilka Wedekind, Liesa Wolf-Baldauf, Minobu Kamo, Jing Zhao, Elif Can, Sarah Keller

**Affiliations:** 1 Diagnostic and Interventional Radiology and Nuclear Medicine, University Medical Center Hamburg-Eppendorf (UKE), Martinistraße 52, 20246 Hamburg, Germany; 2 evidia Group, Alice-Salomon-Platz 2, 12627 Berlin, Germany; 3 St. Luke’s International Hospital, 9-1 Akashi-cho, Chuo- ku, 104-8560 Tokyo, Japan; 4 Department of Radiology, Charité Universitaetsmedizin Berlin, corporate member of Freie Universität Berlin and Humboldt- Universität zu Berlin, Charitéplatz 1, 10117 Berlin, Germany; 5 Institute of Medical Biometry and Epidemiology, University Medical Center Hamburg-Eppendorf (UKE), Hamburg, Germany

**Keywords:** Authorship, Gender, Women, Impact, Career

## Abstract

**Background:**

Career prospects in academic medicine are strongly linked to scientific authorship and this marker has been widely used as an indicator of gender equity in academia. However, direct comparisons of medical disciplines regarding their proportion of female physicians (FP) in different countries are missing. This study examines the gender parity and gender cooperation using first authorships (FA) and senior authorships (SA) of scientific publications in five medical disciplines and six different OECD countries over a 10-year time-trend.

**Methods:**

Articles from three high-impact journals in each of the medical discipline radiology, urology, surgery, gynecology, and pediatrics from the years 2007/8 and 2017/18 were retrospectively reviewed. The gender and affiliation location of the FA and SA of original research articles and reviews were assigned and compared with the proportion of in each discipline for the United States of America, Canada, United Kingdom, France, Germany, and Japan. Mantel-Haenszel test and multinomial logistic regression models were used to calculate differences in proportions of women authors and FP and to assess trends and proportions of FA and SA.

**Results:**

30,803 articles were evaluated. Equally, with rising proportions of FP in all disciplines, the number of women authors increased across years. The shares of women FAs were either significantly higher (urology/surgery/gynecology) or balanced (pediatrics/radiology) compared to the proportion of FP. In contrast, the shares of women SA were balanced only in disciplines with a low proportion of FP (urology and surgery) and otherwise reduced. Women same-gender cooperation was as common as men same-gender cooperation and preferred over a women-led mixed gender cooperation in disciplines where this seemed to be practicable due to the high proportions of FP.

**Conclusion:**

In contrast to FA, a significant disparity persists in SA, particularly in disciplines with a high proportion of FP. The discrepancy between FA and SA may reflect, among others, dropout from an academic career in early or mid-academic levels, for example, due to structural inequality; together with the findings on gender preference in authorship collaborations, this may inform future strategies for promoting equal career advancement for women physicians.

**Supplementary Information:**

The online version contains supplementary material available at 10.1186/s12909-023-04041-6.

## Introduction

Despite a global increase in the number of women physicians (FP) in recent decades and a resulting higher probability for women to start an academic career at a university medical institution, fewer women are still encountered in leading positions in academic medicine [1]. For example, in 2018 in the United States (US), 48% of medical graduates are women but only 25% are full professors, and 18% are department chairs [2]. In the same year, as many as 61.6% of graduates in Germany were women [3] and only 24.4% held a full professorship in 2019 [4]. Viewed from the perspective of gender parity in academic and scientific medicine, equal representation of female authorship in scientific publications is an important measure of equity in academic careers. *Gender parity* here refers to ‘the equal contribution of women and men to different dimensions of life’ and it is executed as a ‘relative equality in terms of numbers and proportions of women and men’ for a given indicator, such as academic authorship or proportion of senior physicians in academic institutions [5]. While in academic medicine gender parity decreases with increasing hierarchical levels, in clinical medicine, including non-university institutions and practices, female proportions are found that are similar to the graduation rate of female medical students. Other medical disciplines have, in part historically, comparably low proportions of women. An academic career in medicine is still considered the highest goal to strive for in many, technologically advanced countries. In contrast to natural science or humanities, a full professorship is in most cases linked to a director or chief physician position. This implies a certain double burden for aspiring physicians, on the one hand outstanding clinical performance and on the other hand, outstanding scientific performance, measured by publications and third-party funding. How successfully young physicians cope with the double burden depends to a non-negligible extent on structural conditions [1]. These include support from superiors and mentors, e.g., through time off from clinical routine, the general workload in the clinic, and the family situation. Academic progress coincides with a period in life when family planning is pending; if structural and private conditions such as childcare and support are not in place, many women drop out of the competition and opt against an academic career and for a more predictable clinical career in the outpatient sector. Aggravatingly, to reach the same position as men, women often must meet higher performance requirements, as shown in a Canadian study on medical-academic positions and the h-index [6, 7]. The importance of government and private support measures has also been demonstrated by the corona pandemic, in which female scientists lost out significantly on publication performance compared to their male counterparts, among others, due to the loss of state childcare [8]. It thus does not appear surprising that despite a positive trend in the development of women graduating from medical schools and junior resident physicians, academic senior positions held by women in particular, and reflected by senior authors (SA) in scientific publications, are still in the minority [9]. To put the share of publications of women first authors (FA) and SA in relation to the available personnel pool of FP in the respective discipline should therefore give an impression of how many women could potentially strive for an academic career and how many, compared to men, finally realize it.

The assessment of scientific authorships in leading international journals is a standard method to evaluate gender parity in academic productivity, performance measurement and recognition [10]. In recent years, a variety of retrospective studies evaluating authorships by gender for certain medical disciplines, such as gynecology, urology, and radiology, have been published [11–18]. In most publications, journals were selected according to their impact factor, so the focus of the results is on the highly ranked journals and less on middle or lower-ranked journals as well as the US context.

The primary objective of this study was, to evaluate the time-trend gender parity in scientific authorships according to medical discipline and county-specific proportions of women physicians in each. The secondary objective was to examine the cooperation between FA and SA based on authorships to conclude possible mentor-mentee connections or preferred research collaborations.

## Methods

An institutional review board exemption was obtained for this retrospective study. For the medical disciplines radiology (rad), urology (uro), surgery (surg), gynecology (gyn), and pediatrics (ped), three representative peer-reviewed journals were selected from the top third of the impact factor rankings using the *Clarivate Analytics Web of Science’s Journal Citation Report*. Care was taken to include journals with a general scope in the respective medical discipline. To analyze the data changes over a definite time frame of ten years, the years 2007/2008 and 2017/2018 were compared. The journals and the impact factor from the years 2007 and 2008, as well as 2017 and 2018, are listed in Table 1.

**Table 1:** Medical journals included in the study

Surgery				
Annals of Surgery (Ann Surg)	7.446	8.460	9.203	9.476
JAMAS surgery (JAMA Surg)	N/A	N/A	8.498	10.668
British Journal of Surgery (Br J Surg)	4.304	4.921	5.433	5.572
**Urology**				
Nature Reviews Urology (Nat Rev Urol)	N/A	N/A	8.089	9.333
Journal of Urology (J Urol)	4.053	3.952	5.381	5.647
BJU International (BJU Int)	2.751	2.704	4.524	4.688
**Gynecology**				
American Journal of Obstetrics and Gynecology (Am J Obstet Gynecol)	2.917	3.453	5.732	6.120
Obstetrics and Gynecology (Obstet Gynecol)	4.282	4.397	4.982	4.965
BJOG – an international journal of obstetrics and gynecology (BJOG: Int J Obstet Gynaecol)	2.666	3.101	4.876	5.193
**Pediatrics**				
Pediatrics (Pediatrics)	4.473	4.789	5.515	5.401
Journal of Adolescent Health (J Adolesc Health)	2.387	2.910	4.098	4.021
Journal of Pediatrics (J Pediatr)	4.282	4.397	N/A	N/A

**Fig. 1:**
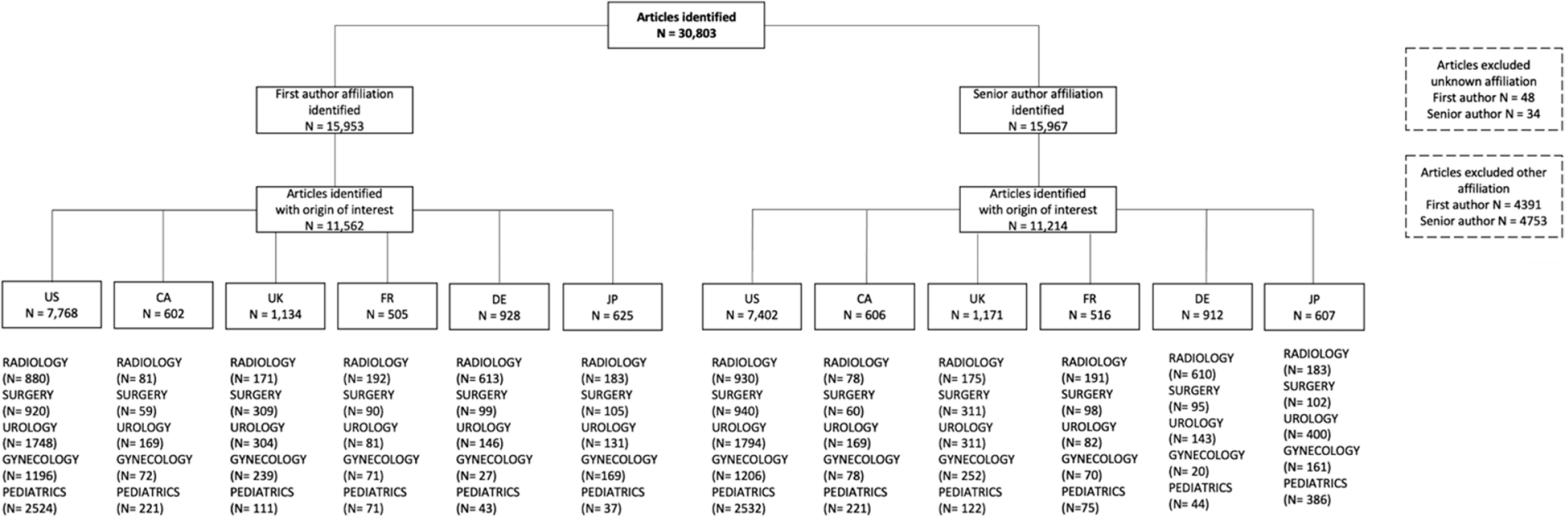
Flow chart of the articles analyzed in the two investigation periods (2007/8 and 2017/18) independent of gender, divided according to the FA/SA and affiliation country of origin. In total *N* = 82 articles with unknown affiliations and *N* = 9144 articles with an affiliation other than the six countries of interest were excluded. Abbreviations: CA, Canada; DE, Germany; FR, France; JP, Japan; UK, United Kingdom; US, United States of America

All publications from these four years were classified according to original research articles, reviews (literature reviews, meta-analyses) and others (e.g., editorials, pictorial essays, letters to the editor). For the analysis, only articles that fell into one of the first two categories were included. The first names and surnames of the FA and SA were assigned a gender for each article. Foreign-language names were evaluated by native speakers (Japanese, Chinese, Vietnamese, Korean). In case of ambiguity (e.g., due to initials of first names), the author names were further evaluated via their institute website, ResearchGate, Google, and software (Gender API; https://gender-api.com). In the case of still unclear gender, the author was classified as unknown. The flowchart in Fig. 1 shows the study protocol.

The number of physicians and the proportion FP in the medical disciplines of countries included in the study (US, CA, United Kingdom (UK), France (FR), Germany (DE), and Japan (JP)) studied were determined via public databases and via direct email contact with the medical associations (Table S1). In the case that no figures were available for the respective year, the next year (e.g., 2009 for 2008) was included. The physician statistics and the proportion of FP were averaged for the years 2007/8, and 2017/18, if both years were available.

### Statistical analysis

Absolute and relative (percentages) numbers were used to describe the data. A multinomial logistic regression model was used to evaluate any differences between percentages of possible FA and SA cooperation (e.g., same-gender men, same-gender women, women-led mixed gender, men-led mixed gender). Percentages for author combinations are given for medical discipline and specific years. Odds ratios (OR) for quantifying the difference between author combinations are reported with 95% confidence intervals, thereby adjusting for medical discipline and year. Mantel-Haenszel test was used to evaluate the difference between the percentage of FP and percentage of women authors per medical discipline, year, FA, and SA with adjustment for the different countries. Due to the explorative study design, all analyses and *P*-values of this study are descriptive, i.e., no adjustment for multiple testing was conducted. Statistic software R (Version 3.5.1; https://www.r-project.org) was used.

## Results

### Overview of articles and FP per country and medical discipline

In total, 30,803 articles were evaluated, out of which 14,773 were original research articles and 1,228 review articles. Of these, the predetermined criteria were met in for 11,562 FA articles and 11,214 SA articles who worked in an institute in the US, UK, CA, FR, DE, or JP. US institutes exhibited the highest number of articles with 7,768 FA and 7,402 SA articles, followed by UK institutes with 1,134 FA and 1,171 SA articles (Fig. 1).

The overall proportion of FP in the countries surveyed varied greatly between countries and medical disciplines. Between 2007/8 and 2017/18, the proportion of FP increased consistently in all disciplines and all countries. The highest total number of physicians was recorded in the US. The proportional increase in FP was most evident in urology and surgery, in some cases doubling over the last decade. More than half of the physicians in gynecology and pediatrics were women (54.3% and 53.1% respectively). In urology and surgery, the proportion of women doctors was lowest (10.9% and 15.3%, respectively) (Table 2).

**Table 2:** Proportion of female physicians (FP) by medical disciplines and year

	Absolute number all gender (percentage female)									
	Radiology		Surgery		Urology		Gynecology		Pediatrics	
	**2007/8**	**2017/18**	**2007/8**	**2017/18**	**2007/8**	**2017/18**	**2007/8**	**2017/18**	**2007/8**	**2017/18**
**US**	27,550 (20.8)	34,793 (23.4)	65,796 (8.8)	67,395 (13.0)	9915 (4.7)	9917 (8.7)	39,665 (43.2)	41,619 **(57.0)**	54,016 **(55.4)**	63,902 **(61.7)**
**DE**	6661 (31.1)	8663 (35.6)	29,325 (14.9)	37,422 (20.8)	4995 (11.0)	6006 (17.1)	16,042 **(54.1)**	18,525 **(67.7)**	11,881 **(51.7)**	14,851 **(59.3)**
**UK**	3392 (34.6)	4495 (38.3)	15,210 (22.8)	17,102 (25.4)	1612 (20.4)	1964 (23.1)	5426 **(58.6)**	6285 **(66.6)**	7625 **(57.5)**	8891 **(63.8)**
**CA**	3392 (25.5)	2558 (31.8)	4279 (11.9)	4491 (22.7)	604 (5.6)	709 (10.7)	1714 (42.9)	2223 **(59.8)**	2228 (48.2)	2598 **(59.8)**
**FR**	7829 (29.5)	8844 (34.8)	9024 (8.2)	10,845 (15.7)	767 (2.7)	1297 (6.3)	7321 **(52.3)**	7836 **(58.7)**	6847 **(60.4)**	8844 **(63.9)**
**JP**	N/A	9700 (24.2)	N/A	39,945 (8.9)	7483 (3.3)	8828 (7.1)	N/A	16,809 (37.2)	12,550 (31.2)	22,314 (36.3)
**Bold print**: Percentage of FP over 50%. _Abbreviations: CA, Canada; DE, Germany; FP, female physicians; FR, France; JP, Japan; UK, United Kingdom; US, United States of America_.										

### Gender parity in scientific authorship in relation to FP

**Fig. 2:**
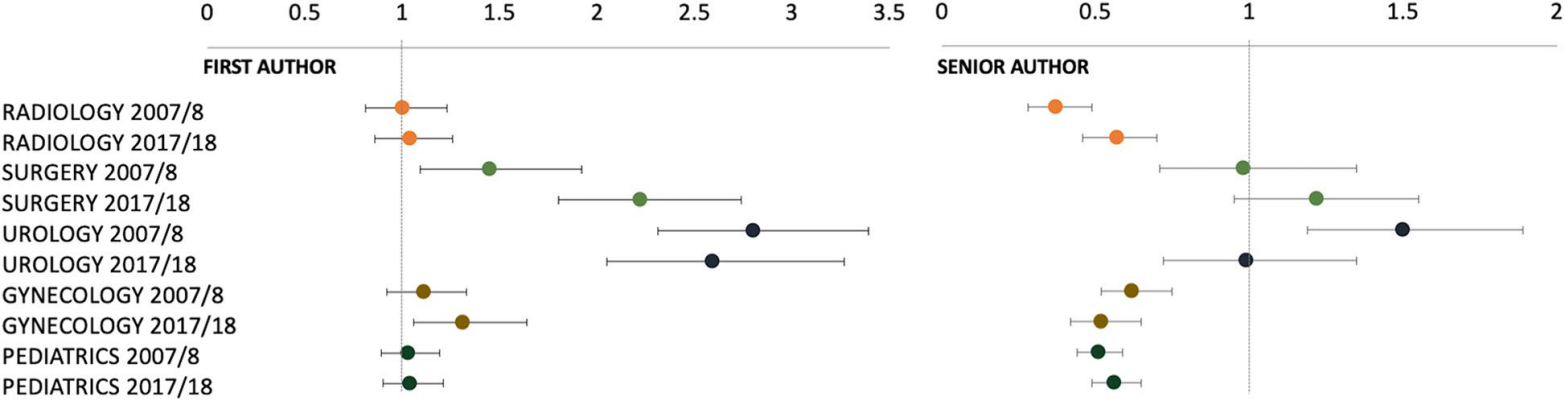
Trend in women publications between 2007/8 and 2017/8 in the different medical disciplines. Compared to the number of FP, the proportion of women FA is significantly increased in surgery and urology, and radiology. The proportion of women SA to FP is balanced or even increased in urology and surgery. Graphs display the odds ratio and 95% confidence intervals for the comparison of proportion women author vs. proportion FP. Results of the Mantel-Haenszel test. Abbreviations: FA, first author; FP, female physician; SA, senior author

The results of gender parity by FA and SA, time point, and medical discipline in conjunction with the proportion of FP are shown in Fig. 2; Table 3.

Gender parity in terms of the proportion of FP was evident in the authorships of women FA across all medical disciplines. The disciplines urology and surgery, with their overall low proportion of FP, showed a disproportionately high proportion of women FA over the last 10 years (OR 1.45–2.80; *P* < 0.0103). In the medical disciplines of radiology and pediatrics, the proportions of FA were balanced in 2007/8 and 2017/18 (OR 1.0–1.04; *P* > 0.05). Gynecology showed a trend from balanced FA shares to disproportionately high shares (OR 1.11; *P* = 0.2852; OR 1.31; *P* = 0.0176). With regard to scientific publications of women SA, a more differentiated picture emerged: In gynecology, pediatrics and radiology, the share of women SA was constantly lower than the share of FP in 2007/8 and 2017/18 (OR 0.37–0.62; *P* < 0.0001). In contrary, women SA were more common (OR 1.50; *P* < 0.0001) or balanced (OR 0.98–1.22; *P* > 0.05) in urology and surgery.

**Table 3:** Proportion of women authors in the medical disciplines and years. The odds ratio (OR) represents the comparison between the percentage of women authors and percentage of female physician (FP) in each specialty

		2007/8					2017/18				
	Author Type	Total Author	Female Author	OR [95% CI]	*P*-Value	Total Author		Female Author	OR [95% CI]	*P*-Value	
**Radiology**	First Author	503.5	129 [25.62]	1.00 [0.81–1.23]	0.9865	523		157 [30.02]	1.04 [0.86–1.26]	0.6954	
	Senior Autor	**515.5**	**59.5 [11.54]**	**0.37 [0.28–0.49]**	**< 0.0001**	**549.5**		**104 [18.93]**	**0.57 [0.46–0.70]**	**< 0.0001**	
**Surgery**	First Author	**365.5**	**61.0 [17.11]**	**1.45 [1.09–1.92]**	**0.0103**	**424.5**		**124.5 [29.33]**	**2.22 [1.80–2.74]**	**< 0.0001**	
	Senior Autor	363	43.5 [11.98]	0.98 [0.71–1.35]	0.9960	435		80 [18.39]	1.22 [0.95–1.55]	0.1245	
**Urology**	First Author	**851.5**	**146 [17.15]**	**2.80 [2.31–3.39]**	**< 0.0001**	**421**		**99 [23.52]**	**2.59 [2.05–3.27]**	**<0.0001**	
	Senior Autor	**875**	**88 [10.06]**	**1.50 [1.19–1.89]**	**0.0005**	432.5		45.5 [10.52]	0.99 [0.72–1.35]	0.9930	
**Gynecology**	First Author	491	225 [45.82]	1.11 [0.92–1.33]	0.2852	**356.5**		**219.5 [61.57]**	**1.31 [1.06–1.64]**	**0.0176**	
	Senior Autor	**512.5**	**167.5 [32.68]**	**0.62 [0.52–0.75]**	**< 0.0001**	**361**		**144.5 [40.03]**	**0.52 [0.42–0.65]**	**< 0.0001**	
**Pediatrics**	First Author	741.5	411.5 [55.50]	1.03 [0.89–1.19]	0.7241	754.5		470.5 [62.36]	1.04 [0.90–1.21]	0.6128	
	Senior Autor	**750.5**	**285.5 [38.04]**	**0.51 [0.44–0.59]**	**< 0.0001**	**760.5**		**360 [47.34]**	**0.56 [0.49–0.65]**	**< 0.0001**	

### Gender parity in author cooperation by medical discipline and year

**Fig. 3:**
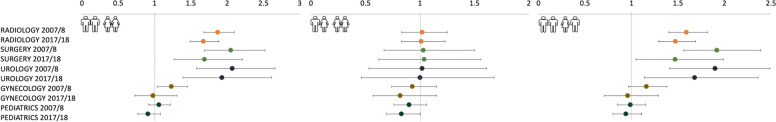
Trend in probabilities for gender-specific author cooperation combined for all countries, separated by medical discipline and year. Presentation of a temporal trend towards greater gender diversity in publication cooperation, with same-gender men cooperation remaining significantly more likely than same-gender women cooperation in radiology, surgery, and urology. On the other hand, higher probabilities of same-gender women cooperation in pediatrics and balanced same-gender cooperation in gynecology are shown. Graphs display the odds ratio and 95% confidence intervals for the comparison of same-gender men authorship vs. same-gender women authorship (left), women-lead mixed-gender authorship (women SA, men FA) vs. same-gender women authorship (middle), same-gender men authorship vs. men-lead mixed-gender authorship (men SA, women FA) (right). Results of multinomial logistic regression

Figure 3 illustrates publication figures regarding a gender-specific author cooperation between FA and SA.

In all medical disciplines, there was a trend towards same-gender women or mixed-gender author collaboration between 2007/8 and 2017/18. However, despite this positive trend, same-gender men collaboration remained much more likely than same-gender women cooperation in radiology, urology, and surgery (OR 1.67–1.93; *P* < 0.0001). In these three disciplines, men-led same-sex collaborations were also more likely than men-led mixed-gender (men SA, women FA) collaborations (OR 1.47–1.68; *P* < 0.0001). Gynecology was the only medical discipline with a balanced probability for either men or women same-gender cooperation in 2017/18 (OR 0.98; *P* > 0.05). In pediatrics, a same-gender women collaboration was even more likely than a same-gender men collaboration (OR 0.91; *P* < 0.0002) in 2017/18. Comparable to subjects with a higher likelihood of men same-sex collaboration, gynecology and pediatrics, medical disciplines with a balanced or higher likelihood of women same-gender collaboration, were less likely to have a women-led mixed-gender (women SA, men FA) collaboration than a same-gender women collaboration (OR 0.82–0.83; *P* < 0.0001).

## Discussion

This study examined the representation of women authorship relative to the number of registered women physicians in five medical disciplines and six OECD countries. The results show that already in the first study period 2007/8 the proportion of women FA, mostly representing physicians in the early stages of an academic career, was either disproportionately high (urology and surgery) or balanced (radiology, gynecology, pediatrics) compared to the proportion of FP in each medical discipline in the countries studied. This pattern remained constant ten years later in 2017/18 or continued to increase in favor of women FA in gynecology and surgery.

In contrast, the proportion of women SA, mostly representing faculty members of middle and senior academic positions, lags the proportion of FP in most disciplines. Interestingly, only urology and surgery, two medical disciplines which have low proportions of FP in general, show to have a balanced to an increased proportion of SA throughout the period studied. This is an interesting finding which, at the beginning of the study, we would have attributed to the disciplines with a high proportion of FP, such as gynecology and pediatrics.

Shifting the focus to disciplines related to women and child health, gynecology and pediatrics have a high proportion of FP (in 2017/18 in all countries except JP over 50%), however, according to our analyses, the proportion of women SA does not correspond to the high proportion of FP in these disciplines. One possible explanation is that the two disciplines attract many FP who see their careers in clinical and outpatient medicine, while FP who might choose a men-dominated discipline such as surgery or urology have generally different expectations and goals regarding their academic careers. On the other hand, this result may also be an expression of structural inequalities in academic medicine that lead to higher dropout rates of female academic physicians after completing residency training or in the middle academic levels.

Regarding publication cooperation in the disciplines, it becomes obvious, comparable to previous literature, that same-gender men author cooperation is still significantly more likely than same-gender women author cooperation. The probability of same-gender men cooperation remains also greater than that of men-led mixed-gender cooperation, which could be interpreted as a preference for a same-gender mentor-mentee relationship. Only in gynecology and pediatrics, two disciplines with a high proportion of FP same-gender women cooperation is equally frequent and even more likely than women-led mixed-gender cooperation, findings that have already been described in previous studies [14]. In contrast to other studies [11], however, we did not see this relationship in the field of urology; here, as in the other two disciplines of surgery and radiology, the probability of same-gender women cooperation was not higher than in women-led mixed-gender cooperation. The findings lead to the conclusion that in medical disciplines with higher gender parity and thus more choice, same-gender cooperation is preferred, whereas in disciplines with gender parity of physicians not yet achieved same-gender women cooperation is probably more difficult to realize because of the low proportion of women senior physicians working in academics. In medical disciplines with a low proportion of FP in senior positions, the preferred same-sex publication collaboration thus risks reducing the number of supporters or mentors to advance the scientific career of junior FP. If the observation of preferred same-gender cooperation is a proxy for gender-specific mentorship, national and institutional efforts should be directed towards advancing women senior faculty positions and creating women networks and mentoring programs.

Besides same-gender mentor-mentee relationships via role models having a positive influence on the academic career of future generations, gender parity in the professional environment has been shown to create a broader range of perspectives, strengths, and backgrounds, which can have a positive impact on productivity and academic progress [19, 20]. It is generally assumed that there is a close academic relationship between the FA and SA that ultimately results in a successful publication. Although we did not examine the seniority of FAs and SAs in detail in this study, we generally assume a junior-senior relationship in the distribution of these author positions. One reason for this is that FA play a greater role than SA for scientific milestones in a medical career, such as a doctoral or postdoctoral degree. Above, it is assumed that SA generally have a more advanced academic career than FA, they lead working groups and have their third-party funds through which they finance the projects. A mentorship is generally understood as a relationship between two people with different levels of advanced experience in the respective field of interest, with knowledge being passed from mentor to mentee. For medical-scientific publications, it is important that the mentor works in the respective field of research and can support this project through his or her knowledge and experience and, if applicable, acquired third-party funding. Although in past years authorships were often directly related to mentoring relationships [11, 12, 14, 17, 18, 21, 22], generalizability has recently been discussed [23]. However, it remains to be emphasized that this publication-oriented mentoring relationship cannot be equated with informal mentoring in all cases. If the results are generalized to a proxy for gender-specific mentorship, they could inform institutional and national strategies to increase women’s senior positions in academia and to implement formal mentorship and networking programs for women, thereby also addressing the component of informal mentoring relationships. It should be emphasized that further evaluation of FA-SA relations, e.g., based on quantitative surveys, could be an interesting option for future studies to illustrate country-specific differences more precisely in distribution.

Another interesting result of this study is that there are large regional differences in terms of authorship and proportion of FP. JP not only has the lowest proportion of women in all medical disciplines studied, but also the lowest proportion of women FA and SA. A Japanese study on the proportion of women in medical academic positions confirms a steady increase in the number of FP between 1995 and 2018, especially assistant professors, but the mid-level faculty, on the other hand, is still clearly underrepresented with 13.1% (associate professors) to 16.8% (lecturer positions) [24]. These results are also in line with a recent study by Kuhlmann et al. [1], which uses examples of large university hospitals in Germany, Austria, the UK, and Sweden to illustrate that women academics in particular are denied advancement to the middle ranks and thus progress in their academic careers, which is also reflected by the low proportion of women SA. Interestingly, this study also found strong associations with country-specific welfare state models that can help women to balance work and career, and which supports one of the theses that, due to still existing structural inequalities in academic medicine, FP are more likely to leave university medicine after completing residency to pursue a purely clinical career.

Another limiting factor in the advancement of women to high academic positions is the amount of third-party funding. In disciplines with many men physicians such as radiology and surgery, studies showed lower chances of women postdoctoral applicants for NIH funding [25, 26], whereas, in the FP-dominated fields gynecology and pediatrics, the percentage of accepted applications from women was higher [27]. This suggests that the acceptance rate may also be influenced by the absolute number of applications from each gender.

This study has some limitations: In this study, the author numbers in the first and last positions are extrapolated to the number of FP in each discipline and country. This introduces some uncertainty because academic or non-academic employment or professional position was not specified. This general approach of the study to examine a high number of academic publications in many different disciplines and countries justifies the study design and serves the purpose of a general overview of the status quo and hypothesis generation for subsequent studies. In addition, comparing author numbers with female academic physicians by career stage does not appear to meet the goal of the study because it does not factor in physicians who interrupted their academic careers early due to structural, personal, or professional barriers. In an ideal academic world with equal opportunity for both genders regardless of family planning and other factors, the number of academically active FP should be equal to the number of academically active male physicians, across all academic positions. Above, this study takes into analysis the FA and SA positions of scientific publications, neglecting the gender distribution of co-authorship. This is based on the following rationale: the rules for co-authorship in a scientific publication vary significantly in different working groups and research collaborations. Co-authorships are also less decisive for the advancement of a scientific career and the attainment of the next academic degree. Moreover, co-authorships are less likely to reflect successful research collaboration between different academic rank levels and/or mentoring relationships. Taken together, the addition of co-authorship analyses would blur the focus and key messages of this study. Some other limitations are that for JP, the proportions of FP for the year 2007/8 could not be determined in the fields of radiology, surgery, and gynecology, which reduces the significance of the information regarding a temporal trend. The study analysis was performed in 2019, including articles published by the end of 2018; due to a large number of articles (> 30,000), some of which involved tedious arithmetic work to identify individual authors, the analysis times were very long. It must be emphasized that this study nevertheless contains the most up-to-date evaluations compared to the currently published literature. For this study, five medical disciplines were examined regarding gender parity in publication performance. The rationale for selecting these five disciplines was to examine a broad diversity of diagnostic, conventional, and surgical specialities of diverse patient clientele that simultaneously exhibit significant differences in the gender distribution of physicians. The comparison of these disciplines, which are very different from the point of view of gender parity, makes it possible to identify and compare influencing factors such as the importance of female senior positions and mentoring ratios more clearly. A pure focus on medical disciplines and sub-disciplines in the field of women’s health may give a different picture and would be interesting to examine in more detail in future studies. Furthermore, the journals of the respective medical specialities were selected based on their impact rankings irrespective of a wide range of subspecialties; a random imbalance in the number of published articles in the discipline-specific subspecialties was not explicitly investigated. However, discipline-specific differences in the gender distribution of different subspecialties were described in various studies [14, 17, 21].

## Conclusion

In conclusion, the increasing trend in the proportion of FP in all medical disciplines studied has been accompanied by an increasing proportion of female FAs, and as a result, in all disciplines, women are publishing at least the same, if not more, than the proportion of FP would indicate. These results raise hope that as the number of scientifically active FP increases, the proportion of female SAs, consistently lowered over the 10 years studied, will also adjust in the coming years. However, in a contrast, the slow increase and still low representation of female SAs are indicative of factors that negatively affect the progression of FP into the academic middle and senior levels. Considered together with the preference for same-gender author collaboration as a possible proxy for a same-gender mentoring relationship, new strategies for targeted career advancement of women physicians at national and institutional levels may emerge, particularly in the transition phase following residency.

## Supplementary Information

**Additional file 1.**

## Data Availability

The datasets used and/or analysed during the current study are available from the corresponding author on reasonable request.
