# Supplementary material for: Gender differences and cooperation in medical authorships - an analysis of the recent ten years in five key medical disciplines
Source: BMC Med Educ. 2023 Jan 27;23:68. doi: 10.1186/s12909-023-04041-6 (PMC9883917; doi:10.1186/s12909-023-04041-6)
Supplement: Supplementary file 1 — Additional file 1. [file 12909_2023_4041_MOESM1_ESM.docx]

Table S1. Reference of the number of female physicians in the different countries and specialties.

| Country | Reference | URL link | Access date |
| --- | --- | --- | --- |
| United States | Association of American Medical Colleges (AAMC) | <https://www.aamc.org/data-reports> | 24 Nov 2018 |
| United Kingdom | National Health Service (NHS) | <https://digital.nhs.uk/data-and-information/find-data-and-publications/supplementary-information/2018-supplementary-information-files/analysis-of-the-representation-of-women-across-the-hospital-and-community-health-services-workforce> | 04 Jan 2019 |
| Germany | Bundesaerztekammer | <https://www.bundesaerztekammer.de/ueber-uns/aerztestatistik/> | 12 Nov 2018 |
| Canada | Canadian Medical Association | <https://www.cma.ca/En/Pages/physician-historical-data.aspx> | 24 Nov 2018 |
| France | Direction de la recherche, des études, de l’évaluation et des statistiques (DREES) | <http://www.data.drees.sante.gouv.fr/ReportFolders/reportFolders.aspx?sCS_referer=&sCS_ChosenLang=fr> | 18 Feb 2020 |
| Japan | Japanese Medical Association | Excel file send via email | 9 Feb 2019 |
